# Hybrid Fe_3_O_4_-Gd_2_O_3_ Nanoparticles Prepared by High-Energy Ball Milling for Dual-Contrast Agent Applications

**DOI:** 10.3390/ijms27020910

**Published:** 2026-01-16

**Authors:** Vladislav A. Mikheev, Timur R. Nizamov, Alexander I. Novikov, Maxim A. Abakumov, Alexey S. Lileev, Igor V. Shchetinin

**Affiliations:** 1 Laboratory of Multifunctional Magnetic Nanomaterials, University of Science and Technology MISIS, 119049 Moscow, Russia; vmikheev@misis.ru (V.A.M.); nizamov.tr@misis.ru (T.R.N.); novikov.ai@misis.ru (A.I.N.); 2 Department of Physical Materials Science, University of Science and Technology MISIS, 119049 Moscow, Russia; magnito@mail.ru; 3 Laboratory of Biomedical Nanomaterials, University of Science and Technology MISIS, 119049 Moscow, Russia; abakumov.ma@misis.ru; 4 Department of Medical Nanobiotechnology, N.I. Pirogov Russian National Research Medical University, 117513 Moscow, Russia

**Keywords:** magnetite, spinel, gadolinium oxide, high-energy ball milling, nanoparticles, wet milling, magnetic resonance imaging, dual contrast agents, relaxivity, magnetic properties

## Abstract

This work investigates the feasibility of synthesis hybrid x Gd_2_O_3_ + (100 − x) Fe_3_O_4_ nanoparticles using the scalable method of high-energy ball milling for dual-contrast magnetic resonance imaging applications. Comprehensive studies of the structure, magnetic and functional properties of the hybrid nanoparticles were conducted. It was found that the milling process initiates the transformation of the cubic phase c-Gd_2_O_3_ ($Ia\bar{3}$) into the monoclinic m-Gd_2_O_3_ (C2/m). Measurements of the magnetic properties showed that the specific saturation magnetization of the Fe_3_O_4_ phase is substantially reduced, which is a characteristic feature of nanoparticles due to phenomena such as surface spin disorder and spin-canting effects. The transmission electron microscopy results confirm the formation of hybrid Fe_3_O_4_-Gd_2_O_3_ nanostructures and the measured particle sizes show good correlation with the X-ray diffraction results. A comprehensive structure–property relationship study revealed that the obtained hybrid nanoparticles exhibit high r_2_ values, reaching 160 mM^−1^s^−1^ and low r_1_ values, a characteristic that is determined primarily by the presence of a large fraction of Gd_2_O_3_ particles with sizes of ≈30 nm and Fe_3_O_4_ crystallites of ≈10 nm.

## 1. Introduction

Early diagnosis of malignant tumors remains one of the most pressing healthcare issues worldwide [1]. Various imaging methods are used for this purpose, such as ultrasound, computed tomography (CT), and magnetic resonance imaging (MRI). Among all these methods, MRI possesses a number of distinct advantages [2,3,4], such as its inherent safety (absence of ionizing radiation), high spatial resolution, and the ability to obtain three-dimensional reconstructions of tissues and organs. MRI is widely used for the visualization and diagnosis of diseases of the central nervous system, the musculoskeletal system, and a number of internal organs, including the assessment of dynamic processes occurring within tissues and organs. In this regard, MRI is of high social significance, especially for the purpose of early disease diagnosis. Due to its physical principle, which is based on the relaxation of protons in a magnetic field, MRI provides comprehensive information on both the anatomical structure and the physicochemical state of tissues [5]. Contrast agents are currently used to enhance diagnostic information. They allow for a more accurate diagnosis of tumor lesions by increasing the contrast resolution of MRI images [6]. Currently, contrast agents can be divided into two primary groups: “positive” (T_1_-contrast agents that enhance the signal and make specific areas of the MRI image appear brighter) and “negative” (T_2_-contrast agents that attenuate the signal and render regions darker).

T_1_-contrast agents include, for example, gadolinium-based compounds (mainly gadolinium chelate complexes) [7] and manganese-based agents [8]. The administration of T_1_-contrast agents during an MRI examination results in a decrease in the spin-lattice relaxation time. The efficacy of T_1_-contrast agents is fundamentally due to the large magnetic moment of their paramagnetic ions [9].

The introduction of T_2_-contrast agents during MRI leads to a decrease in the spin–spin relaxation time. Iron oxide-based nanoparticles (maghemite, magnetite) have proven to be excellent T_2_-contrast agents [10,11]. Furthermore, by varying the size of the nanoparticles as well as modifying their shape, it is possible to control (increase or decrease) the T_2_-relaxation rate by altering the T_2_-relaxation time of water protons surrounding the contrast agent [12]. Thus, even at identical concentrations of the contrast agent, it is possible to obtain different contrast effects. Moreover, a number of contrast agents have been developed based on magnetic iron oxide nanoparticles, the surface of which is modified with biocompatible and non-toxic organic ligands such as polyethylene glycol (PEG) [13], human serum albumin (HSA) [14], and also copolymer molecules such as Pluronic^®^ [15], which contain both hydrophilic and hydrophobic parts in their structure. Molecules of such ligands serve a dual function: on the one hand, they prevent the aggregation of nanoparticles in solution, and on the other hand, they increase their average circulation time in the bloodstream by masking them from recognition and clearance by macrophages of the reticuloendothelial system. Furthermore, polyethylene glycol and copolymers based on it are clinically approved substances included in various cosmetic and medicinal products [16]. In both cases, the use of contrast agents leads to an increase in contrast within the examined tissues and provides additional, extremely important information that is necessary for disease diagnosis, especially in the early stages.

However, all of the above-mentioned contrast agents possess inherent drawbacks that limit their widespread use. Thus, “positive” gadolinium-based contrast agents are generally associated with toxicity concerns (during their application, there exists a risk of inducing nephrogenic systemic fibrosis in patients with impaired renal function, especially in elderly patients) [17,18]. Moreover, research into new classes of compounds of this type aimed at improving their functional properties has resulted in newly synthesized substances typically exhibiting rather low solubility in aqueous media (for example, some currently used Gd chelates are very rapidly cleared from the circulatory system) [19], which again limits their application. Iron oxide nanoparticles used as “negative” MRI agents, on the other hand, are not highly toxic [20] and can be excreted through physiological pathways [21,22]. A significant limitation is that this applies only to nanoparticles, as larger particles administered intravenously may cause vascular embolization [23]. Furthermore, the lack of inherent tissue specificity, as well as instances of low background intensity and the frequent confusion of their signals with those originating from various endogenous formations (such as hemorrhages, calcium deposits, iron accumulations, fat, blood clots, and air bubbles) also limit their application [24].

Given the above, the development of a new generation of contrast agents free from these drawbacks is a highly pressing and relevant task. Moreover, dual T_1_-T_2_ contrast agents (possessing simultaneously high values for both longitudinal (r_1_) and transverse (r_2_) relaxivities, alongside a low r_2_/r_1_ ratio) appear particularly promising in this regard; their use would potentially allow for a significant increase in the diagnostic efficiency and accuracy of MRI [25].

Currently, one of the prominent approaches that allows the production of such advanced materials is the synthesis of composite nanostructures [26] or nanoparticles of complex compounds doped with specific magnetic elements [27]. Indeed, the concept of Fe_3_O_4_-Gd_2_O_3_ composites is well-established, and significant progress has been made in this field, as demonstrated by several studies reporting promising agents [28,29,30,31,32]. For instance, in [28] developed ultrasmall hybrid nanoparticles for T_1_-weighted imaging, while [29] reported PMAO-coated Gd_2_O_3_/Fe_3_O_4_ composites with high dual-mode efficiency. Other innovative strategies include the synthesis of core–shell structures [30], or use of composites [31], and the creation of ultrasmall Fe_3_O_4_ nanoparticles [32]. However, a common challenge among these advanced synthesis routes is their reliance on multi-step wet-chemical methods (e.g., thermal decomposition, co-precipitation, hydrothermal synthesis), which are often complex, difficult to reproduce on a large scale, and require precise control over reaction parameters (temperature, pH, precursor injection rates). To address these limitations and provide a robust, scalable alternative, we employed a straightforward and industrially viable method of high-energy ball milling for the preparation of hybrid x Gd_2_O_3_ + (100 − x) Fe_3_O_4_ nanoparticles. This mechanochemical approach offers distinct advantages: it is a single-step, solvent-mediated process that eliminates the need for complex precursor chemistry and high-temperature reactions. It inherently allows for excellent homogenization of components and is readily scalable from laboratory to industrial production volumes. In this context, the aim of this study was to investigate the specifics of obtaining Fe_3_O_4_-Gd_2_O_3_ hybrid nanoparticles using this scalable high-energy milling method, to conduct a comprehensive study of their structure, magnetic and functional properties, and to establish the relationship between the synthesis parameters, structure and properties of the obtained materials.

## 2. Results and Discussion

**Figure 1:**
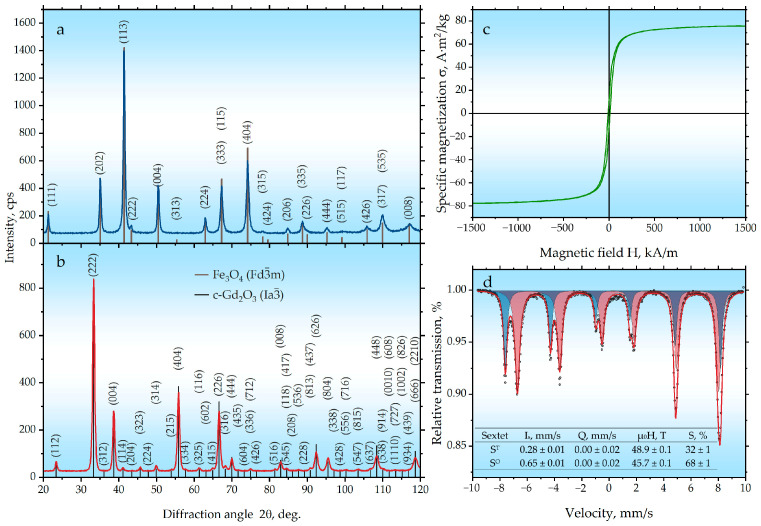
(**a**) XRD pattern of the synthesized Fe_3_O_4._ (**b**) XRD pattern of the Gd_2_O_3_ nanoparticles synthesized by co-precipitation. (**c**) Magnetic hysteresis loop of the Fe_3_O_4_ sample measured at room temperature. (**d**) Room-temperature Mössbauer spectrum of the Fe_3_O_4_ sample.

According to X-ray diffraction (XRD) analysis (Figure 1a, Table 1), the ball-milling of iron powder with water for 25 h leads to the formation of an Fe_3_O_4_ oxide (H1_1_, $Fd\bar{3}m$) with an inverse spinel structure, characterized by a lattice parameter a = 0.8396 nm and a crystallite size of 23.7 nm. This result is in good agreement with the data reported in [33,34,35,36]. Measurements of the magnetic properties (Figure 1c, Table 1 and Table 2) for the sample obtained from milled iron confirm the formation of magnetite. Typical values of specific saturation magnetization are ≈92 A·m^2^/kg for magnetite [37,38] and ≈220 A·m^2^/kg for iron [38,39]. The obtained value of 75.8 A·m^2^/kg confirms the absence of the initial metallic iron in the synthesized sample. The observed reduction in magnetization may be associated with the nanoparticle nature of the product, where reduced magnetic anisotropy leads to a canting of the magnetic moments on the particle surface, a phenomenon that is well-documented in the literature [40,41].

According to the Mössbauer spectroscopy data (Figure 1d, Table 1), the spectrum of the sample obtained from milled iron is described by two components: a sextet (S^T^) with an isomer shift of 0.28 mm/s and a sextet (S^O^) with an isomer shift of 0.65 mm/s. These components correspond to the two nonequivalent positions of iron ions in the tetrahedral (A-site) and octahedral (B-site) coordination environments, respectively. The relative area ratio of these components is close to 1:2, which is typical for stoichiometric magnetite. This finding corroborates the data obtained from both XRD and magnetic property measurements.

The results of the phase analysis for the Gd_2_O_3_ sample, which was synthesized by co-precipitation followed by annealing at 1000 °C for 1 h (Figure 1b, Table 1), showed the presence of the cubic modification of c-Gd_2_O_3_ ($Ia\bar{3}$) with a crystallite size of ≈30 nm. This is in good agreement with the data reported in [42,43].

**Figure 2:**
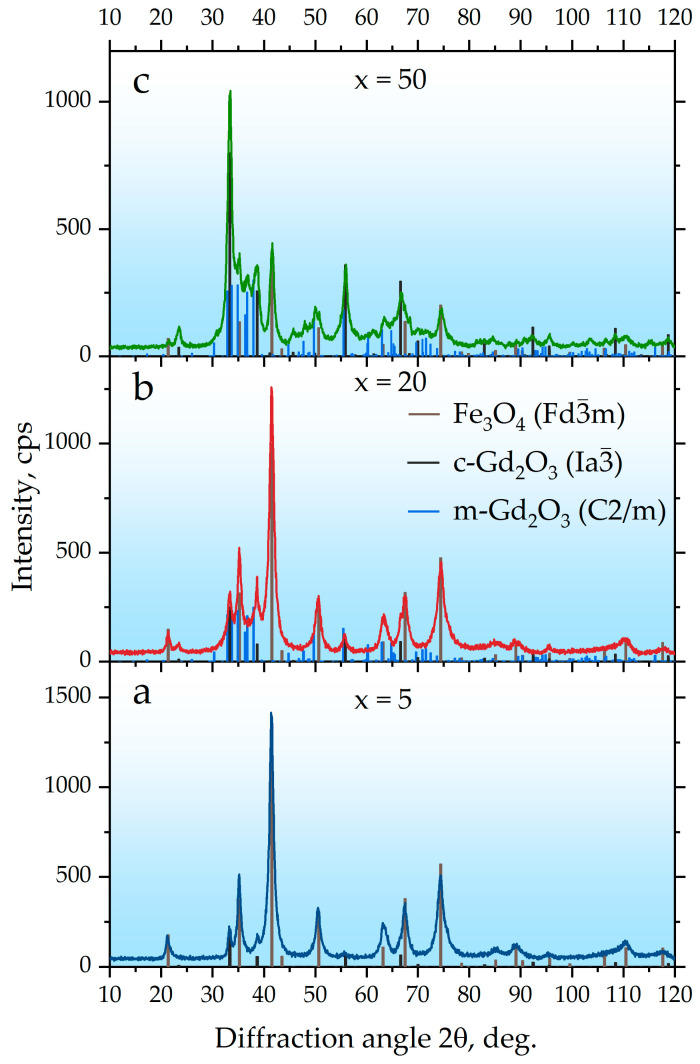
XRD patterns of the hybrid x Gd_2_O_3_ + (100 − x) Fe_3_O_4_ nanoparticles after 100 h of high-energy ball milling; (**a**) x = 5, (**b**) x = 20, and (**c**) x = 50.

XRD analysis of the milled x Gd_2_O_3_ + (100 − x) Fe_3_O_4_ mixtures (Figure 2, Table 1) showed that the component contents are close to the specified values and differ only slightly, remaining within the margin of error. During the joint milling of the mixtures for 100 h, a decrease in the crystallite sizes of the Fe_3_O_4_ ($Fd\bar{3}m$) phase is observed for all samples with x = 5, 10, 20, 30, and 50, from 23.7 nm down to a range of 9.5 to 10.2 nm. The crystallite sizes obtained after milling for 50 h vary only within the experimental error. The lattice parameters of the Fe_3_O_4_ ($Fd\bar{3}m$) phase also decrease after milling the mixture for 50 h. In the samples with x = 5 and 10, according to the phase analysis, the cubic modification of c-Gd_2_O_3_ ($Ia\bar{3}$) is present, with the crystallite size of this phase decreasing very slightly from 29.7 nm to 25.1–25.8 nm after milling, as do its lattice parameters. In the samples with x = 20 to 50, in addition to the cubic modification of gadolinium oxide, the monoclinic m-Gd_2_O_3_ (C2/m) is also detected, which is the thermodynamically stable phase under normal conditions [44,45]. It is worth noting that the obtained crystallite sizes for m-Gd_2_O_3_ (C2/m) have values close to the crystallite size values for magnetite and fluctuate within the range from 8.3 to 9.1 nm. Thus, it can be concluded that the grinding process provokes a phase transformation in the gadolinium oxide particles from the cubic to the monoclinic modification. The monoclinic modification of m-Gd_2_O_3_ (C2/m) has a rather complex XRD pattern, and at low concentrations and with very small particle sizes, its diffraction peaks can become broadened and of low intensity, potentially explaining why it is not observed in the x = 5 and 10 samples due to the limited sensitivity of the technique. Thus, the extended milling successfully produces a nanocrystalline composite where Fe_3_O_4_ crystallites are approximately 10 nm in size.

**Figure 3:**
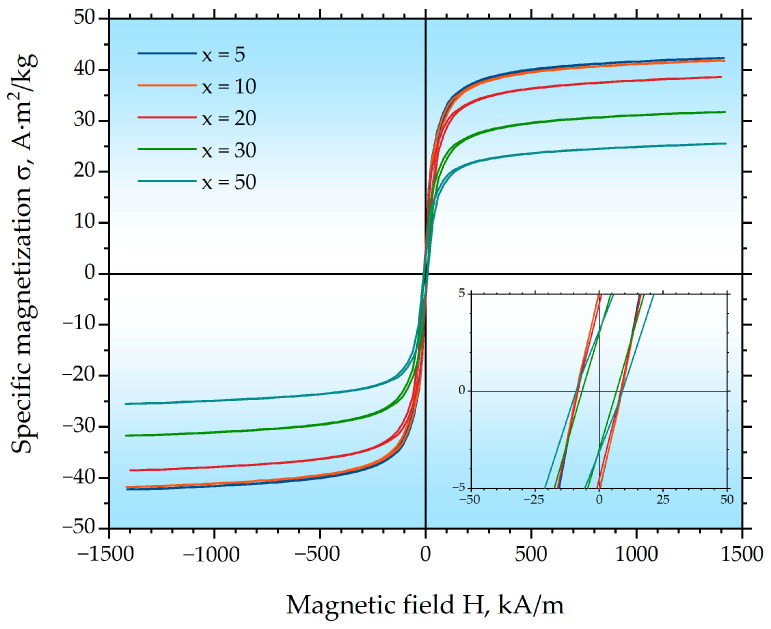
Magnetic hysteresis loops of the milled hybrid x Gd_2_O_3_ + (100 − x) Fe_3_O_4_ nanoparticles, measured at room temperature after 100 h of high-energy ball milling.

Measurements of the magnetic properties of the milled x Gd_2_O_3_ + (100 − x) Fe_3_O_4_ mixtures (Figure 3, Table 2) showed that during the milling the coercivity decreases for all samples, which is most likely due to the formation of a significant amount of superparamagnetic particles. This hypothesis is supported by the XRD data, which indicate an average Fe_3_O_4_ ($Fd\bar{3}m$) crystallite size of approximately 10 nm (Table 1). The size range of 10–20 nm is considered to be the interval for the transition of magnetite to the superparamagnetic state [46,47]. The observed decrease in the specific saturation magnetization is primarily associated with the reduction in the weight fraction of Fe_3_O_4_ ($Fd\bar{3}m$) with increasing x. However, a recalculation based on the actual weight fraction of Fe_3_O_4_ ($Fd\bar{3}m$) gives a specific saturation magnetization value of σ_s_ ≈ 40–44 A·m^2^/kg, which is significantly lower than the values characteristic of pure bulk magnetite and also lower than that of the initial Fe_3_O_4_ nanoparticles (75.8 A·m^2^/kg) with a larger crystallite size (23.7 nm). This further reduction in magnetization is directly correlated with the decrease in the Fe_3_O_4_ crystallite size down to approximately 10 nm after milling with Gd_2_O_3_, as determined by XRD (Table 1). These low obtained values of specific saturation magnetization are characteristic of nanoscale magnetic materials and are attributed to the enhanced contribution of surface spin disorder and spin-canting effects in smaller nanoparticles [48,49,50].

**Figure 4:**
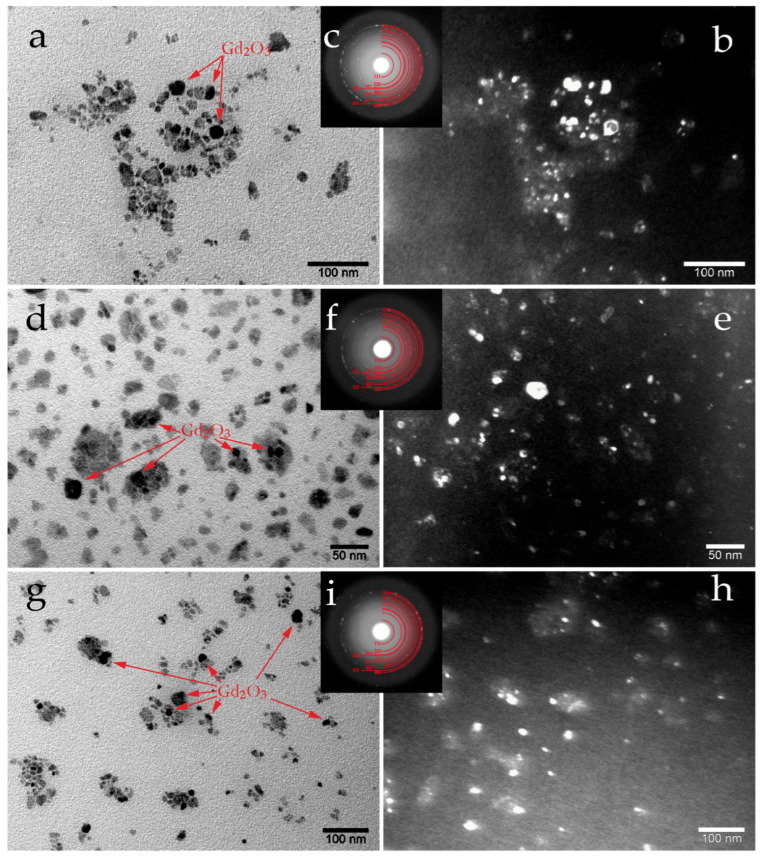
TEM images of the hybrid nanoparticles for compositions x = 5, 20, and 50. (**a**,**d**,**g**) Bright-field images; (**b**,**e**,**h**) Dark-field images; (**c**,**f**,**i**) Selected area electron diffraction (SAED) patterns. Darker particles of Gd_2_O_3_ are indicated by red arrows.

To confirm the formation of nanoparticles, transmission electron microscopy (TEM) studies were conducted (Figure 4). The bright-field image of the x = 5 sample (Figure 4a) shows the presence of very small particles, thereby confirming the data obtained from both XRD and magnetic property measurements. The selected area electron diffraction (SAED) images (Figure 4c) corresponds well to the Fe_3_O_4_ ($Fd\bar{3}m$) phase, showing solid Debye rings with occasional distinct bright spots. The solid rings are indicative of nanoparticles formation, while the isolated bright spots may be attributed to texturing or a preferential orientation of some particles due to magnetic interaction. The dark-field image enables a quantitative assessment of the particle size distribution; however, the bright-field image reveals relatively rare large and dark particles. Due to absorption contrast, these darker particles are identified as c-Gd_2_O_3_ ($Ia\bar{3}$), and their size correlates very well with the XRD data (Table 1). The TEM images of samples x = 20 (Figure 4d–f) and x = 50 (Figure 4g–i) are similar in nature, but they show a gradual reduction in the size of the dark particles, which is consistent with the XRD data. According to XRD, at larger x values, a phase transformation occurs from the cubic c-Gd_2_O_3_ ($Ia\bar{3}$) to the monoclinic m-Gd_2_O_3_ (C2/m), accompanied by a decrease in particle size from 29.7 nm down to 8–9 nm (Table 1). According to the TEM data (Figure 4), it is clearly seen that the magnetite and gadolinium oxide particles are in close contact, which confirms the successful production of hybrid Gd_2_O_3_-Fe_3_O_4_ nanoparticles.

**Figure 5:**
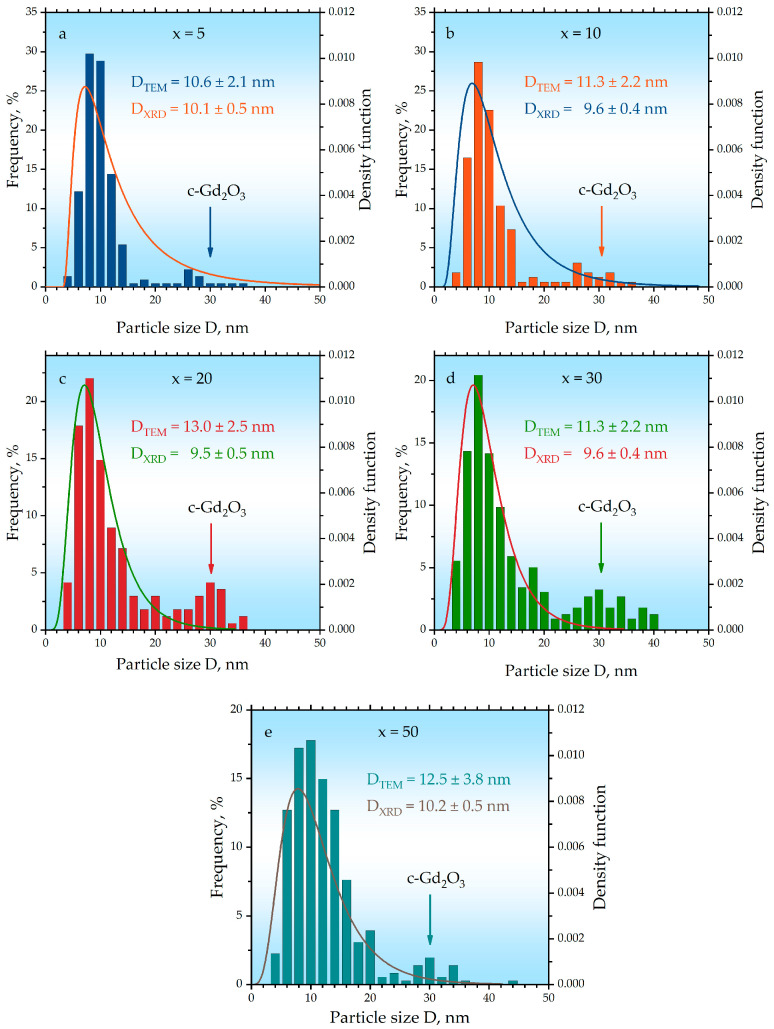
Particle size distribution analysis for the hybrid nanoparticles samples x = (**a**) 5, (**b**) 10, (**c**) 20, (**d**) 30, (**e**) 50. Histograms represent the distribution obtained from dark-field TEM image. The lognormal curves represent the size distribution of the Fe_3_O_4_ phase derived from XRD line broadening analysis using the fundamental parameters method. The values D_TEM_ and D_XRD_ denote the average particle sizes determined from TEM histograms and XRD Rietveld refinement, respectively.

The particle size distributions determined by complementary techniques are summarized in Figure 5. Histograms represent the distributions obtained from statistical analysis of dark-field TEM images (n ≥ 350 measurements per sample). These histograms consistently reveal a bimodal distribution across all compositions. For instance, in the sample with x = 5, the dominant component, attributed to Fe_3_O_4_, has an average size of D_TEM_ = 10.6 ± 2.1 nm, which is in excellent agreement with the crystallite size derived from XRD Rietveld refinement (D_XRD_ = 10.1 ± 0.5 nm, Table 1). The secondary component of larger particles (~30 nm) corresponds to the cubic c-Gd_2_O_3_ phase. This assignment is further supported by the lognormal size-distribution curves (Figure 5) modeled from XRD line broadening analysis using the fundamental parameters method, which describe the Fe_3_O_4_ ($Fd\bar{3}m$) phase well. The quantitative agreement between D_TEM_ and D_XRD_ for the magnetite phase confirms the reliability of both methods. With increasing gadolinium content (x), the relative proportion of the secondary ~30 nm component increases up to x = 20 and then stabilizes, a trend consistent with the quantitative phase analysis (Table 1). Furthermore, both TEM histograms and XRD-derived distribution functions show a broadening of the main ~10 nm peak at higher x values. This broadening is attributed to the increasing contribution of the small monoclinic m-Gd_2_O_3_ (C2/m) particles, whose crystallite size (8.3–9.1 nm, Table 1) is comparable to that of Fe_3_O_4_.

**Figure 6:**
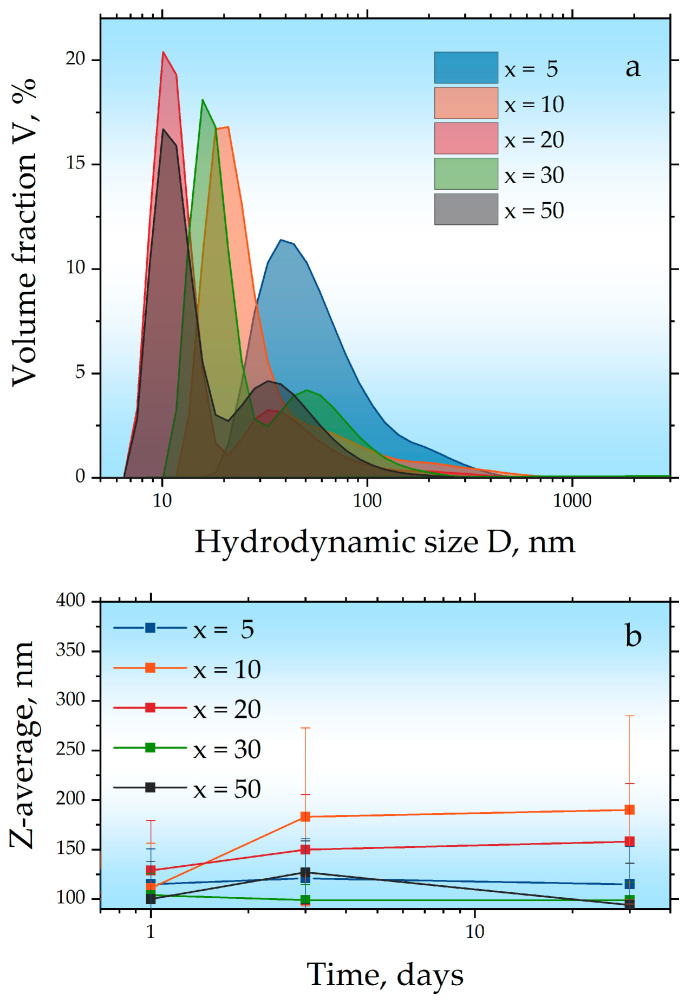
Hydrodynamic diameter (by volume) of the samples measured by DLS after hydrophilization with Pluronic F127 (**a**) and the corresponding Z-average evolution over time (**b**).

In the next step, the synthesized samples with different x values were coated with Pluronic^®^ F-127 copolymer and studied using dynamic light scattering (DLS) (Figure 6a). The DLS size distributions were evaluated using the volume-weighted representation. According to the DLS data, the polymer coating process for sample x = 5 resulted in the formation of aggregates with sizes of approximately 30 nm. The aggregation observed during the coating process may be attributed to strong magnetic dipole–dipole interactions arising from the high content of the magnetic phase in this particular sample. In the remaining samples, aggregation was considerably less pronounced. A bimodal size distribution has previously been reported for nanoparticles hydrophilized with Pluronic F127 [27], as this polymer can adsorb onto the surfaces of individual nanoparticles as well as their aggregates. For all samples except x = 5, the DLS data also revealed the presence of a fraction with sizes of ≈30 nm, which is consistent with the previous results and corresponds to the cubic c-Gd_2_O_3_ ($Ia\bar{3}$) phase. The colloidal stability of the hydrophilized samples was assessed by monitoring the Z-average—an intensity-weighted mean hydrodynamic size—using DLS measurements performed one day, three days, and 35 days after hydrophilization (Figure 6b). For all samples, the measured sizes remained within the standard deviation, indicating no statistically significant change in Z-average over the studied period, despite a slight increasing trend.

**Figure 7:**
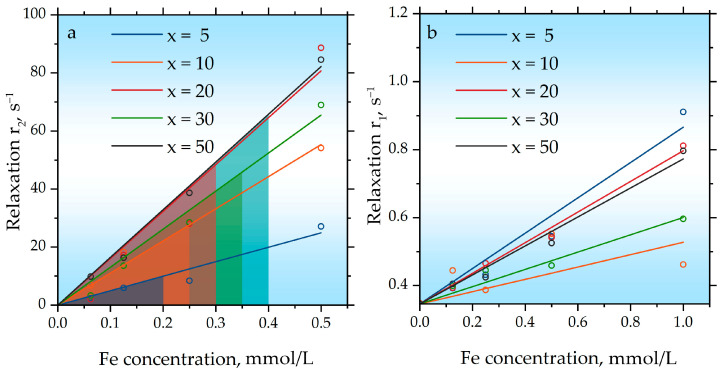
Results of samples relaxivity measuring (**a**) r_2_ and (**b**) r_1_ for hybrid x Gd_2_O_3_ + (100 − x) Fe_3_O_4_ nanoparticles.

Relaxivity measurements for the samples show that they possess a high r_2_ value (Figure 7a, Table 2). This enhancement is explained by the presence of the magnetic moment of the nanoparticles, which induces strong magnetic field inhomogeneity around the nanoparticles, thereby disrupting the spin coherence of water protons and accelerating their transverse relaxation. However, the r_2_ values for sample x = 5 were lower than for all other samples, which is attributed to partial aggregation during the polymer coating process and a consequent reduction in the effective surface area of the nanoparticles available for interaction with water protons. The highest r_2_ values were obtained for samples x = 20 (164 ± 5 mM^−1^s^−1^) and x = 50 (161 ± 17 mM^−1^s^−1^), which is due to the fact that these samples contained the finest nanoparticle fraction. The lack of a decrease in r_2_ with increasing x is explained by the fact that the obtained dependences were plotted against the concentration of iron ions; therefore, for samples with higher x, the effective concentration of magnetite nanoparticles remained constant. The obtained r_2_ values for samples x = 20 and x = 50 are at a high level [51,52].

All synthesized hybrid nanoparticle samples exhibited low r_1_ values, ranging from 0.2 to 0.5 mM^−1^s^−1^, falling within the margin of error. The obtained low r_1_ is a direct consequence of the large size (≈30 nm) of the gadolinium oxide particles. The efficacy of a T_1_ contrast agent is governed by two primary parameters for inner-sphere relaxation: the number of water molecules directly coordinated to the paramagnetic ion and their exchange rate. In our system, the large c-Gd_2_O_3_ ($Ia\bar{3}$) particles possess a low surface-to-volume ratio, which drastically limits the fraction of Gd^3+^ ions that are surface-exposed and accessible for direct coordination with water molecules [53]. Consequently, the effective number of water molecules directly coordinated to the paramagnetic ion value for the entire nanoparticle is extremely low. Furthermore, even for the surface Gd^3+^ ions, the water exchange kinetics are likely suboptimal. In large, dense oxide matrices, the exchange of water molecules in the inner coordination sphere can be significantly slowed down compared to small molecular chelates. Thus, the combination of a severely limited number of accessible Gd^3+^ sites and potentially slow water exchange rates results in the negligible r_1_ observed in our hybrid nanoparticles, despite the high paramagnetic moment of Gd^3+^. We attribute the persistence of these large Gd_2_O_3_ particles to the significant difference in the mechanical properties (hardness, brittleness) between Gd_2_O_3_ and Fe_3_O_4_, which likely leads to non-optimal and inhomogeneous energy transfer during the high-energy ball milling process, preventing the efficient comminution of the gadolinium oxide phase. Future work will focus on optimizing the milling parameters and precursor particle sizes to achieve more effective co-grinding and a substantial reduction in the c-Gd_2_O_3_ ($Ia\bar{3}$) particle size, which is expected to unlock the T_1_ contrast potential of these hybrid systems. Moreover, the hydrophilized nanoparticles possess a dual stabilizing shell: an inner hydrophobic oleic acid layer and an outer Pluronic F127 layer, whose hydrophobic polypropylene oxide segment adsorbs onto the surface while the polyethylene oxide chains extend into the aqueous phase. This additional hydrophobic barrier further limits the access of water molecules to Gd ions, which should also contribute to the reduced r_1_ values. While the observed aggregation in DLS and the low r_1_ values highlight areas for improvement, they are inherent outcomes of the chosen synthesis method in its current, non-optimized form.

## 3. Materials and Methods

In this study, gadolinium oxide nanoparticles were synthesized using a coprecipitation method. All reagents, including gadolinium nitrate hexahydrate Gd(NO_3_)_3_·6H_2_O (99.99%) and ammonium hydroxide NH_4_OH, were purchased from Sigma-Aldrich (Merck KGaA, Darmstadt, Germany). Two grams of gadolinium nitrate were placed in a 500 mL beaker, 100 mL of bidistilled water was added, and the mixture was stirred for 1 h at 50 °C. Ammonium hydroxide NH_4_OH was then added dropwise to the nitrate solution. The resulting precipitate was filtered and dried, and then calcined for 1 h at 1000 °C.

Magnetite nanoparticles were obtained by mechanochemical synthesis with the addition of water. The method is described in more detail in [52]. Fe powder (Sigma–Aldrich, 99.5%) and bidistilled water were used. Synthesis was carried out in a PM400 planetary ball mill (Retsch GmbH, Haan, Germany) for 25 h at a jar rotation speed of 400 rpm. Ten grams of Fe powder and 5 g of water were placed in the milling jar. The iron powder to milling media mass ratio was 10:200. Hybrid Fe_3_O_4_-Gd_2_O_3_ nanoparticles were synthesized by ball milling in the non-polar solvent 1-octadecene with oleic acid as a surfactant. For each composition, 2 g mixtures of the Gd_2_O_3_ and Fe_3_O_4_ powders obtained in the first stage were prepared such that the oxide mass ratio was x% Gd_2_O_3_ and (100 − x)% Fe_3_O_4_, where x = 5, 10, 20, 30, and 50. The resulting mixtures, weighing 2 g each, were placed in milling jars; 1-octadecene (95%) and oleic acid (90%) were also added. The mass ratio of milling media, powder, 1-octadecene, and oleic acid was 200:2:30:2. The resulting mixture was subjected to high-energy ball milling in a PM400 planetary ball mill (Retsch GmbH, Haan, Germany) for 100 h with jars rotation speed of 400 rpm. These specific milling parameters were selected based on our previous optimization study, which established them as optimal for achieving effective homogenization and the target structural properties in magnetic nanoparticles [11]. After milling, the product was collected and diluted threefold with 1-butanol. The resulting precipitate was isolated, washed twice with ethanol, and subsequently redispersed in dichloromethane to obtain a final concentration of 8 mg/mL.

The phase transfer from organic to aqueous media was performed following a previously reported protocol with minor modifications [54]. Our previous studies on analogous iron oxide nanoparticle systems have demonstrated that this specific surface chemistry results in excellent biocompatibility [11]. Furthermore, a recent study from our group on nanoparticles with a directly comparable core–shell–copolymer architecture, synthesized via a similar route, confirmed their low toxicity in detailed in vitro assays [55]. A 0.25 mL aliquot of the nanoparticle dispersion in CH_2_Cl_2_ (8 mg/mL) was four-fold diluted with toluene and mixed with an equal volume of an aqueous Pluronic F127 solution (25 mg/mL). The mixture was sonicated until a uniform gray emulsion formed. The emulsion was then centrifuged at 1000× *g*, after which the upper clear organic layer was replaced with an equal volume of water. The mixture was sonicated again and subjected to a second centrifugation step at 12,000× *g*. The resulting precipitate was redispersed in water to yield the final hydrophilic nanoparticle dispersion.

Structural characterization of the synthesized powders was carried out by X-ray diffraction (XRD) on a Rigaku MiniFlex600 (Rigaku, Tokyo, Japan) instrument. The measurements were conducted in Bragg–Brentano geometry with CoKα radiation (λ = 0.179012 nm), using a K_β_ filter and a D/teX linear detector (Rigaku, Japan) for data collection. To extract quantitative phase information, lattice constants, and substructure parameters, the obtained diffraction patterns were refined by the Rietveld method. This refinement was performed within the PDXL-2 (Rigaku, Tokyo, Japan, ver. 2.8.4.0) software environment, utilizing the ICDD PDF-2 (ICDD, Newtown Square, PA, USA) database. The intrinsic physical broadening of the diffraction peaks was isolated from instrumental effects by performing a calibration with a LaB_6_ standard. Finally, distributions of particle sizes were modeled directly from the diffraction data using the fundamental parameters approach available in the PDXL-2 package.

The local electronic environment of iron atoms in the samples was probed by ^57^Fe Mössbauer spectroscopy. Measurements were recorded at 298 K in transmission geometry with constant acceleration mode, employing an MS-1104Em spectrometer (Research Institute of Physics, SFU, Rostov-on-Don, Russia) equipped with a ^57^Co(Rh) radioactive source. Energy scale calibration of all spectra was performed using a metallic α-Fe foil as a reference. Spectral fitting and the extraction of hyperfine parameters were conducted with the Univem MS software package (version 2.1.6.73) from the same institution.

To evaluate the magnetic hysteresis behavior, measurements were carried out at room temperature using a commercial vibrating sample magnetometer VSM-250 (Dexing Magnet Tech, Xiamen, China). The applied external field was varied up to 1592 kA/m to trace the complete magnetization loops.

Microstructural analysis of the synthesized materials was performed by transmission electron microscopy (TEM) on a JEM-1400 (JEOL, Tokyo, Japan) instrument operated at 120 kV. Imaging was conducted in both bright-field and dark-field configurations, supplemented by selected area electron diffraction (SAED). For TEM analysis, a representative sample was prepared by dispersing the powder in ethanol via ultrasonic treatment, followed by drop-casting the resulting colloidal suspension onto a carbon-supported copper grid. To obtain statistically reliable particle size distributions, measurements were preferentially taken from dark-field images due to their superior capability to resolve individual nanoparticles within aggregates. The iTEM software (Olympus, Tokyo, Japan, ver. 5.1) was employed to measure the horizontal diameter of each particle, with a minimum of 350 measurements collected per sample to ensure statistical significance.

Iron quantification in the solutions was achieved via the ferrozine colorimetric method. Absorbance readings were taken at a wavelength of 560 nm using a Thermo Scientific Multiskan GO (Thermo Fisher Scientific Corporation; Waltham, MA, USA) spectrophotometer. The concentration of Fe^3+^ ions was subsequently calculated by interpolation from a pre-established standard calibration curve.

The hydrodynamic diameter distribution of the nanoparticles in suspension was assessed by dynamic light scattering (DLS) using a Zetasizer Nano ZS system (Malvern Panalytical Ltd., Malvern, UK). All DLS measurements were performed at 25 °C using disposable plastic cuvettes.

Measurements of the T_1_ and T_2_ relaxation times were performed using a ClinScan magnetic resonance imaging scanner (Bruker BioSpin MRI GmbH, Ettlingen, Germany) operating at a magnetic field strength of 7 T and a temperature of 23 °C. Aqueous solutions of the samples with Fe^3+^ concentrations of 0.125, 0.25, 0.5, 1.0, and 2.0 mM were prepared.

For T_1_ measurements, an inversion-recovery spin-echo sequence was employed, with an echo time TE = 6,5 ms and inversion times TI ranging from 50 to 2500 ms. The signal recovery curves were fitted to a mono-exponential function to extract the longitudinal relaxation time (T_1_) for each concentration.

For T_2_ measurements, a multi-spin-echo sequence was used with a repetition time (TR) of 10,000 ms and a set of echo times (TE) ranging from 8 to 240 ms. The signal decay curve for each concentration was fitted to a mono-exponential function (Equation (1)) [27].

(1) $$S_{i}=\;S_{0}e^{\left(-\frac{TE}{T}\right)}$$

where $S_{0}$—is the signal at the initial time;

$S_{i}$—is the signal at time TE.

Graphs of the time dependence 1/T on particle concentration were plotted in Excel. Relaxivity was defined as the slope of this linear dependence.

## 4. Conclusions

In this study, we systematically investigated the specifics of using high-energy ball milling as a scalable method for producing hybrid x Gd_2_O_3_ + (100 − x) Fe_3_O_4_ nanoparticles, where x = 5, 10, 20, 30, 50. The results of phase analysis confirm the formation of the Fe_3_O_4_-Gd_2_O_3_ composite; at larger x values, the milling process initiates the transformation of the cubic modification c-Gd_2_O_3_ ($Ia\bar{3}$) into the monoclinic m-Gd_2_O_3_ (C2/m). Measurements of the magnetic properties show a decrease in the coercivity for all samples, which is associated with the formation of a large fraction of superparamagnetic particles. The specific saturation magnetization of the Fe_3_O_4_ phase for all samples is at the level of σ_s_ ≈ 40–44 A·m^2^/kg, which is significantly lower than the values for pure, bulk magnetite. The obtained low values of specific saturation magnetization confirm the formation of nanoparticles, since nanoscale materials are characterized by misorientation of magnetic moments on the particle surface and spin-canting effects. The TEM results confirm the formation of hybrid Fe_3_O_4_-Gd_2_O_3_ nanoparticles, and the measured particle sizes are in good agreement with the X-ray diffraction results. The hybrid nanoparticles exhibit high r_2_ values of 160 mM^−1^s^−1^ and low r_1_ values, which is determined primarily attributed to the large fraction of gadolinium oxide particles with sizes around 30 nm.

## Figures and Tables

**Table 1:** Results of XRD analysis of samples.

Sample	Phase Content, %			Phase Parameters				
	Fe_3_O_4_ ($Fd\bar{3}m$)	c-Gd_2_O_3_ ($Ia\bar{3}$)	m-Gd_2_O_3_ (C2/m)	a_Fe3O4_, nm (0.0002)	D_Fe3O4_, nm	a_c-Gd2O3_, nm (0.0002)	D_c-Gd2O3_, nm	D_m-Gd2O3_, nm
Fe_3_O_4_	100	-	-	0.8396	23.7 ± 0.5	-	-	-
Gd_2_O_3_	-	100	-	-	-	1.0815	29.7 ± 1.2	-
x = 5	96 ± 3	4 ± 1	-	0.8370	10.1 ± 0.5	1.0809	25.8 ± 1.2	-
x = 10	92 ± 3	8 ± 2	-	0.8372	9.6 ± 0.5	1.0811	25.1 ± 1.2	-
x = 20	84 ± 3	9 ± 2	7 ± 2	0.8370	9.5 ± 0.5	1.0811	25.7 ± 1.2	9.1 ± 0.5
x = 30	73 ± 3	12 ± 2	15 ± 2	0.8368	9.6 ± 0.5	1.0807	26.7 ± 1.2	8.7 ± 0.5
x = 50	53 ± 3	15 ± 2	32 ± 3	0.8372	10.2 ± 0.5	1.0813	27.1 ± 1.2	8.3 ± 0.5

**Table 2:** Results of measuring the properties of samples.

Sample	Coercivity H_c_, kA/m	Specific Remanent Magnetization σ_r_, A·m^2^/kg	Specific Saturation Magnetization σ_s_, A·m^2^/kg	r_1_, mmol/L/s	r_2_, mmol/L/s
Fe_3_O_4_	12 ± 1	11.9 ± 0.1	75.8 ± 0.5	-	-
x = 5	9 ± 1	5.1 ± 0.1	42.3 ± 0.3	0.5 ± 0.1	50 ± 5
x = 10	9 ± 1	5.0 ± 0.1	41.8 ± 0.3	0.2 ± 0.1	111 ± 5
x = 20	8 ± 1	4.5 ± 0.1	38.6 ± 0.2	0.5 ± 0.1	161 ± 17
x = 30	7 ± 1	3.0 ± 0.1	31.7 ± 0.2	0.3 ±0.1	131 ± 8
x = 50	9 ± 1	3.1 ± 0.1	25.5 ± 0.2	0.4 ± 0.1	164 ± 5

## Data Availability

The data presented in this study are available in the article.
